# Characterization of different alginate lyases for dissolving *Pseudomonas aeruginosa* biofilms

**DOI:** 10.1038/s41598-020-66293-2

**Published:** 2020-06-10

**Authors:** Núria Blanco-Cabra, Bernhard Paetzold, Tony Ferrar, Rocco Mazzolini, Eduard Torrents, Luis Serrano, Maria LLuch-Senar

**Affiliations:** 1 Bacterial infections and antimicrobial therapies, Institute for Bioengineering of Catalonia (IBEC), The Barcelona Institute of Science and Technology (BIST), Barcelona, Spain; 2 S-Biomedic N.V. Turnhoutsweg 30 2340, Beerse, Belgium; 3 EMBL/CRG Systems Biology Research Unit, Centre for Genomic Regulation (CRG), The Barcelona Institute of Science and Technology, Dr Aiguader 88, Barcelona, 08003 Spain; 4 Universitat Pompeu Fabra (UPF), 08003 Barcelona, Spain; 5 Institució Catalana de Recerca i Estudis Avançats (ICREA), 08010 Barcelona, Spain

**Keywords:** Applied microbiology, Biofilms

## Abstract

Aggregates of *Pseudomonas aeruginosa* form a protective barrier against antibiotics and the immune system. These barriers, known as biofilms, are associated with several infectious diseases. One of the main components of these biofilms is alginate, a homo- and hetero-polysaccharide that consists of β-D-mannuronate (M) and α-L-guluronate (G) units. Alginate lyases degrade this sugar and have been proposed as biotherapeutic agents to dissolve *P. aeruginosa* biofilms. However, there are contradictory reports in the literature regarding the efficacy of alginate lyases against biofilms and their synergistic effect with antibiotics. We found that most positive reports used a commercial crude extract from *Flavobacterium multivorum* as the alginate lyase source. By using anion exchange chromatography coupled to nano LC MS/MS, we identified two distinct enzymes in this extract, one has both polyM and polyG (polyM/G) degradation activities and it is similar in sequence to a broad-spectrum alginate lyase from *Flavobacterium sp*. S20 (Alg2A). The other enzyme has only polyG activity and it is similar in sequence to AlyA1 *from Zobellia galactanivorans*. By characterizing both of these enzymes together with three recombinant alginate lyases (a polyM, a polyG and a polyM/G), we showed that only enzymes with polyM/G activity such as Alg2A and A1-II’ (alginate lyase from *Sphingomonas sp*.) are effective in dissolving biofilms. Furthermore, both activities are required to have a synergistic effect with antibiotics.

## Introduction

Biofilms are complex and dynamic structures formed by different pathogens that cause chronic persistent and recurrent infections. Among 65–80% of human infections are associated with biofilms^1–4^. They are especially frequent in pulmonary infectious diseases like cystic fibrosis (CF), chronic obstructive pulmonary disease (COPD), bronchiectasis and ventilator-associated pneumonia (VAP). The challenge of treating biofilm infections is the increased resistance of the bacteria within the biofilm to antimicrobial agents and host defense mechanisms^5^. The metabolic activity of the bacterial cells is low, resulting in slow-growing cells with radically downregulated cell division rates^6^. Therefore, antibiotics such as β-lactams, which are only active against dividing cells, are not very efficient at eradicating biofilm infections. These diseases are very difficult to manage therapeutically, as the minimum effective bactericidal concentration of antibiotics for biofilm eradication *in vivo* is impossible to reach without causing adverse effects and renal and/or hepatic injury. Moreover, many of the pathogenic bacterial strains are antibiotic resistant. In particular, critically ill patients who are intubated and on mechanical ventilation are at greater risk of developing VAP^7^. *P. aeruginosa* and *Staphylococcus aureus* are the primary causative pathogens of biofilm-associated pulmonary infections^7,8^.

When these bacteria grow in biofilms, they form an extracellular matrix that acts as a barrier against antibiotics and the host immune system^9^. In the lung of CF patients, *P. aeruginosa* generally transitions to a mucoid phenotype characterized by the overproduction of the exopolysaccharide alginate^10^. Alginate is the most abundant extracellular matrix polysaccharide and consists of β-D-mannuronate (M) and α-L-guluronate (G) as monomeric units. These units can be linked in three different kinds of blocks, poly β-D-mannuronate (polyM), poly α-L-guluronate (polyG) or the heteropolymer (polyM/G)^11^. The alginate is O-acetylated at the C-2 and/or C-3 positions in mannuronate residues^12–14^. The monomer composition and the acetylation change depend on the strains and the carbon source. Alginate is one of the most extensively studied *P. aeruginosa* virulence factors and it is associated with persistence in the chronically inflamed airway^15–17^. Therefore, when trying to identify possible drug targets, many studies have focused on the alginate production pathway or the degradation of alginate by enzymes^18–23^.

Biochemical characterization of different alginate lyases^24–34^ has revealed different polyM and polyG activities. The enzymes are classified into seven polysaccharide lyase (PL) families (PL5, 6, 7, 14, 15, 17, and 18) in the Carbohydrate-Active enzymes (CAZY) database^35^. Alginate lyases from family PL7 have been widely studied, and the crystal structures of several PL7 alginate lyases are solved^36–38^. Structural analysis shows that these lyases share a common β-sandwich fold consisting of two β-sheets, in which conserved amino acid residues compose a deep active cleft that is covered by two flexible lid loops^36–38^. This family comprises both endolytic and exolytic alginate lyases.

The substrate specificities of PL7 alginate lyases are also diverse, including polyG-specific, polyM-specific, and bifunctional enzymes. One example of a bifunctional PL7 alginate lyase from *Sphingomonas sp*. A1 is A1-II’^39^. With respect to the PL5 family, AlgL is a 40-kDa poly-β-D-mannuronate lyase from *P. aeruginosa* that preferentially degrades deacetylated polyM *via* a β-elimination reaction, resulting in disaccharides and trisaccharides as its major products^40,41^. Purified alginate lyases from *P. aeruginosa* strains (AlgL) have polyG/M activity and have been shown to dissolve *P. aeruginosa* PAO1 strain biofilms^25^. Alginate lyases A1-I (PL5 + 7 family), A1-II (PL7 family) and A1-III (PL5 family) from *Sphingomonas sp*. strain A1 can produce di- and trisaccharides as major final products from alginate^42^. In addition, an oligoalginate lyase of this bacterium can degrade alginate oligosaccharides into their respective constituent monosaccharides^43^. A1-I is active against acetylated and non-acetylated alginates. A1-II, on the other hand, prefers polyG and non-acetylated alginate. A1-III efficiently liquefies polyM and acetylated alginates produced by mucoid cells.

Alginate lyase treatment has been shown to reduce viscosity both in cultures of clinical isolates and in CF sputum^29^; it strips biofilms from abiotic surfaces^22,44^, enhances phagocytosis of *P. aeruginosa* by human immune cells^20,23,45^, and improves the efficacy of various antipseudomonal antibiotics^18,19,45,46^. However, although multiple studies have reported that the biofilm inhibitory concentration (BIC) of antibiotics against *P. aeruginosa* biofilm cultures is lowered when combined with alginate lyase activity^18–21,47^, it was also shown that synergy between tobramycin and the A1-III and AlgL enzymes is completely decoupled from their catalytic activity^47^. This raises the question of whether it is their enzyme activity that is important for dissolving biofilms. Moreover, whilst different studies using commercial crude extracts containing alginate lyase activity have been shown to dissolve biofilms^18–20,23^, recent studies have shown that the recombinant AlgL enzyme does not show a significant antibiofilm effect; its catalytic activity against alginate and acetylated alginate is not sufficiently high^48^. Thus, a careful reexamination of the alginate lyases responsible for the enzymatic activities present in these extracts could clarify whether these proteins can really be used to dissolve *P. aeruginosa* biofilms and reduce the required dose of antibiotics in CF patients.

Here, we purified five alginases. Four belong to the PL7 family: Alg2A and AlyA1 (both related to the ones found in the crude Sigma extract), A1-II’ and A1-II; and one to the PL5 family (A1-III). We have characterized their activities against polyG, polyM and polyM/G substrates. We also tested their capacity to dissolve biofilms of two *P. aeruginosa* strains (PAO1 *wt* and PAO1Δ*mucA*) and evaluated their synergistic effect when used in combination with the antibiotic ciprofloxacin^49^. The efficacy of this fluoroquinolone in biofilms has been previously assessed by showing that antibiotic resistance of *P. aeruginosa* and *S. aureus* is critically increased during coculture biofilm growth^50^.

## Results

### Isolation and identification of the alginate lyase activities from the crude extract of *Flavobacterium multivorum*

**Figure 1:**
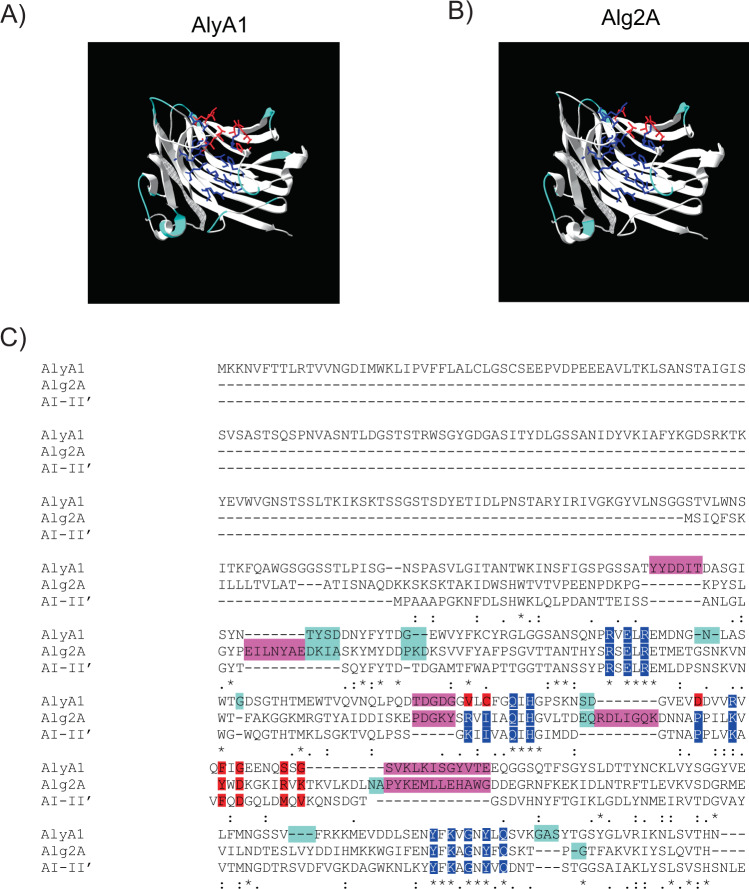
Activity of the Sigma extract fractions obtained after purification: (**A**) activity measured by the halo assay. We show images of the negative control (c-, no halo) and the halo formed with one representative fraction B2 of the Sigma extract (in blue, surrounding the disk). Scale bar corresponds to 10 mm (**B**) alginate lyase activity measured aafter 4 h of incubation and (**C**) SDS gel of the purified fractions that show activity. In the figure, a representative plot of two different experiments is shown.

The crude extract of *Flavobacterium multivorum* from Sigma (A1603 Sigma) was first fractionated using anion exchange and gel filtration chromatography. Then, the enzymatic activity of the different fractions was examined in a halo test done with the *P. aeruginosa* PAO1 *wt* cells (HT, Fig. 1A; Material and methods) and in an activity assay with brown seaweed alginate as the substrate (time point of 4 h; Fig. 1B). With both methods, we identified two separate activities in samples covering the range of fractions from B2 to B5 (Fig. 1A and B). The B2 and B5 fractions, as well as the surrounding ones, were analyzed by SDS gel electrophoresis (Fig. 1C). An upper band of approximately 40 kDa was enriched in fraction B5-B4 and a lower band of about 30 kDa was enriched in fractions B3 to B1, both being consistent across two different samples fractionated the same way.

Both bands were analyzed separately by mass spectrometry (MS) after trypsin and chymotrypsin digestions (Table 1 and Tables S1 to S8). As the source of the extract was unknown, identified spectra were assigned to peptide sequences by performing a *de novo* search^51^. To find protein sequences that had regions of similarity to the peptides found by MS, we used Basic Local Alignment search Tool (BLAST)^52^. BLAST search revealed that the peptide sequences from the lower SDS band were found in five orthologue alginate lyases (Tables S1 to S4). Peptides from the upper band were found in an alginate lyase (*Zobellia galactanivorans* (AlyA1); PL7 family)^35^ and in two proteins with no assigned function (hypothetical proteins; NCBI reference sequences: WP_012026046.1 and WP_012026879.1) (Tables S5 to S8). Alg2A protein from *Flavobacterium sp*. S20 (PL7 family^35^); was selected as representative of the five alginate lyases detected in the lower band since its sequence contained the higher number of MS unique peptides (Fig. S1). These results suggested that enzymes similar to Alg2A and/or AlyA1, as well as proteins belonging to the two hypothetical protein families, could be responsible for degrading *P. aeruginosa* alginate biofilms.

**Table 1:** Mass Spectrometry results of the two bands of the SDS gels run after the purification steps.

Protease used for the digestion	Lower Band		Upper Band	
	Nr of Peptides	Nr of Proteins	Nr of Peptides	Nr of Proteins
Trypsin	529	157	134	73
Chymotrypsin	80	45	188	61

The proteins extracted from the bands were digested with trypsin and chymotrypsin proteases. The table shows the number of peptides identified in each experiment and the Nr of proteins associated after Blast alignment.

### Sequence analysis of AlyA1 and Alg2A

**Figure 2:**
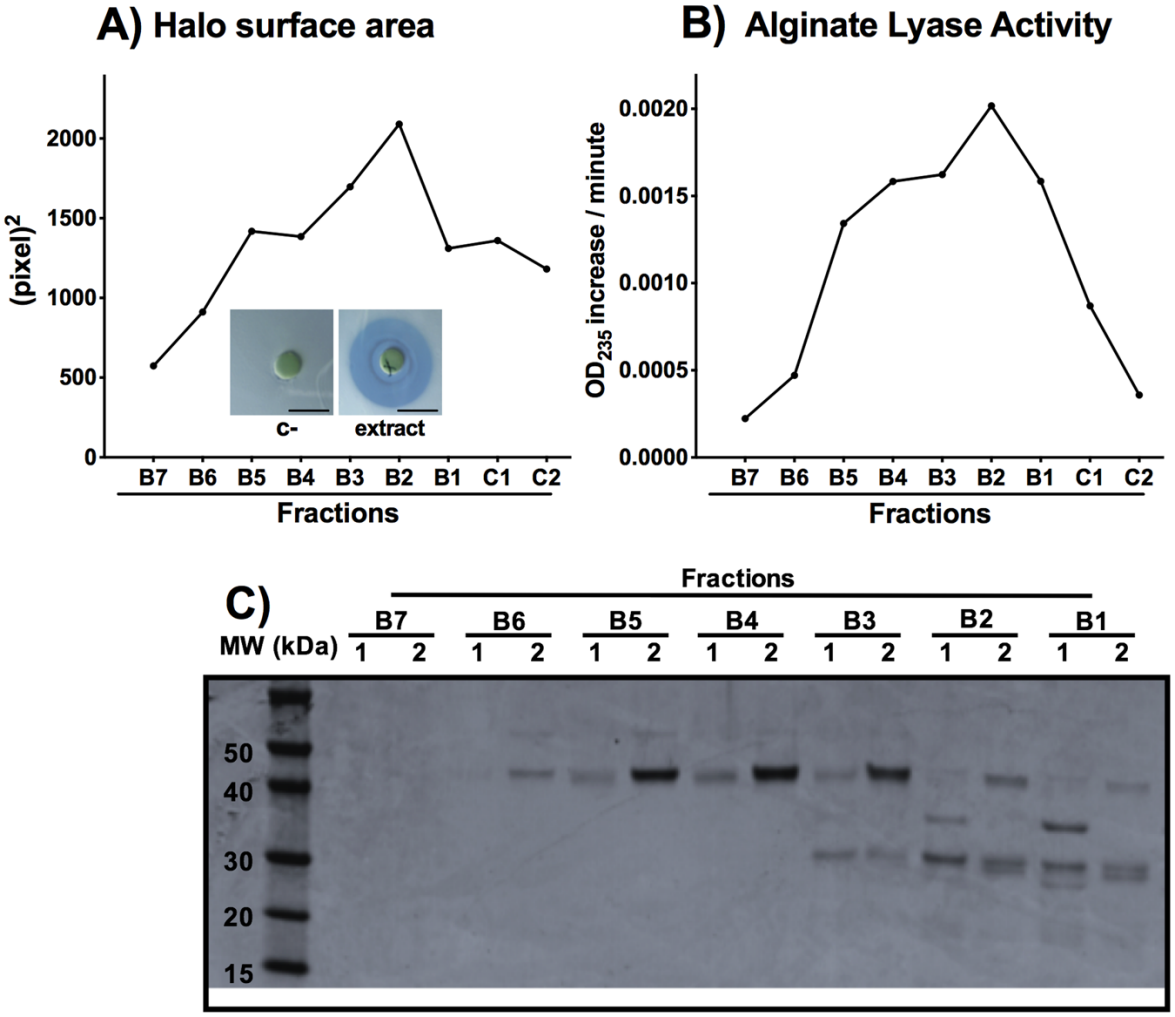
Modelling of AlyA1 and Alg2A based on A1-II’ (2CWS) crystal structure. (**A**,**B**) Models of protein structures of AlyA1 and Alg2A, respectively. Loops involved in the interaction with the substrate are labeled in light blue. In dark blue we show the conserved residues in the cavity close to the putative active site. In red, non-conserved residues of the cavity. (**C**) ClustalW alignment of AlyA1 (WP_013992548.1), A1-II’ and Alg2A (AEB69783.1) protein sequences. Shadowed in dark blue are the residues that form the conserved substrate cavity and in red the important but non-conserved residues of this cavity. Loops important for substrate binding are labelled in light blue. Highly conserved regions described for PL7 family are marked with an asterisk. In bright pink, we show the unique aa sequences present in Alg2A or AlyA1 sequences.

Alg2A (AEB69783.1) contains the highly conserved regions described for the PL7 family: (LIV)-XXWX-(LVI)-(TQN)LP, R-(SVA)-ELRE, NW, (IVLM)(IVLM)(AG)QIH, and YFKAG-X-G-Y-X-Q (Fig. S1), which compose the cavity of a jelly-roll β-sandwich structure where it is assumed to bind a suitable substrate (Fig. 2B). Alg2A has a QIH sequence in its catalytic center, which is a hallmark of polyM/G lyases^53^. It also contains the conserved YFKAGNYFQ motif that is essential for catalysis. The closest alginase in sequence which crystal structure has been determined (2 CWS) is the A1-II’ alginate lyase from *Sphingomonas sp. A1*^54^.

Regarding AlyA1 (WP_013992548.1), sequence alignment shows that it is also a member of the PL7 family and it contains the same elements as Alg2A (Fig. 2C) plus an extra domain of the family 32 (CBM32) that is required for carbohydrate binding^55^. Moreover, AlyA1 contains the QIH sequence that is characteristic of polyM/G alginases. However, it was classified in the SF3 subfamily of the PL7 family, which in theory, should only hydrolyze polyG substrates.

In the Fig. 2C we show the sequence alignment of Alg2A, AlyA1 and A1-II’. Alg2A has four longer loops than AlyA1 – three of which are located around the predicted substrate-binding site (Fig. 2A) – suggesting that this enzyme could recognize or bind to a different polymer structure. With respect to the residues that line the cavity, the conserved ones were associated with the putative active site, but the residues on one side are quite divergent (Fig. 2C-labeled in red). This reinforces the idea that although the catalytic mechanism of A1-II’ and Alg2A must be the same, Alg2A could recognize polymer structures of a different composition. Sequence analysis and comparison with A1-II’ alginate lyase shows a similar picture for AlyA1. The loops suroounding the cavity in the jelly roll where the substrate should bind are of different length and sequence than Alg2A, and the residues surrounding the active site are conserved with those at one end being different (Fig. 2C-labeled in red). In this case the number of loops and residues that are different from the crystal structure is larger than Alg2A.

### Characterization of the alginate-degrading activity of the identified proteins

**Figure 3:**
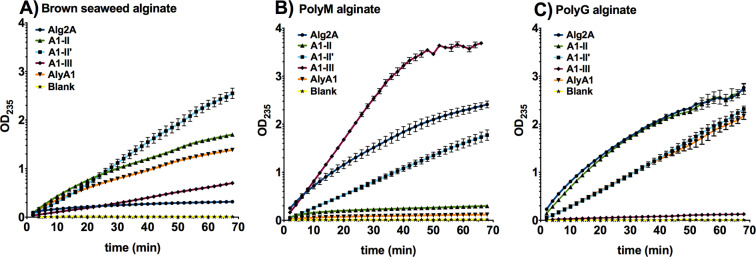
Enzyme activity against different substrates. Alginate lyase enzyme kinetic activity against different substrates: (**A**) brown seaweed alginate, (**B**) polyM alginate and (**C**) polyG alginate. Error bars represent the standard deviation of three different replicates. The blank sample was obtained by adding buffer instead of enzyme and it was subtracted in all the samples.

Although we cloned both hypothetical proteins, we were only able to express and purify WP_012026046.1. Regarding the two alginate lyases (Alg2A (AEB69783.1) and AlyA1 (WP_013992548.1)) identified as possible orthologues of the alginate lyases from the extract, we were able to express and purify both. We also included other alginate lyases, namely A1-II’, A1-II (both PL7 family), and A1-III (PL5 family), to compare the activity of different enzymes by measuring the degradation of different substrates: brown seaweed, polyM and polyG alginate (Fig. 3; Table 2 and Table 3). Alginate lyase activity was determined by following the increase in absorbance at 235 nm due to the formation of a carbon-carbon double bond at the end of the product generated from lyase-mediated cleavage of alginate. We also determined Kcat and Km constants for the different recombinant proteins with PolyG and PolyM substrates (Table 3; Fig. S2). No activity was found for the hypothetical protein (WP_012026046.1) against any of the studied substrates (data not shown).

**Table 2:** Slope values of the linear equations from the activity curves against different substrates for different enzymes.

	Brown seaweed alginate		PolyM		PolyG	
	Slope	r2	Slope	r2	Slope	r2
Alg2A	0.0069	0.969	0.0523	0.995	0.064	0.9936
A1-II	0.0389	0.9965	0.0084	0.9303	0.0651	0.9988
A1-II′	0.0328	0.9985	0.0263	0.9999	0.0299	0.999
A1-III	0.088	0.9995	0.0834	0.9998	0.0024	0.9824
AlyA1	0.0304	0.9906	0.0034	0.9604	0.0312	0.999

R-squared (r2) is the coefficient of determination, or the coefficient of multiple determination for the linear regression.

**Table 3:** Km and Kcat values of different enzimes in presence of PolyM and PolyG substrates.

	PolyG			PolyM		
	Kcat	Km (mg/ml)	Kcat/Km	Kcat	Km (mg/ml)	Kcat/Km
AlyA1	19.68	3.083	6.38			
A1-II	20.55	1.866	11.01			
A1-II′	7.339	1.281	5.73			
A1-III	0.4732	1.248	0.38	85.37	5.36	15.94
Alg2A	18.13	5.531	3.28			

The two polyM/G alginases, A1-II’ and Alg2A, showed both polyG and polyM activity, but although having similar residues in the catalytic pocket, Alg2A showed more activity against both alginates than A1-II’. The specificity constant (Kcat/Km) calculated for each enzyme shows that A1-II’ is more specific for PolyG than Alg2A. Regarding PolyM substrate, we could determine the Kcat/Km specificity constant only for the A1-III enzyme, since in the case of the other proteins, saturation by substrate was not reached under the different concentrations tested (Fig. S2). Moreover, A1-II’ exhibited also activity against brown seaweed alginate, whereas Alg2A did not. AlyA1 has also the QIH sequence characteristic of polyM/G alginate lyases, but lacks activity against polyM alginate and revealed a similar activity to A1-II, displaying both enzymatic activities against polyG alginate and brown seaweed alginate. Regarding A1-III, it showed a great polyM activity a low activity against brown seaweed alginate and without any enzymatic activity against the polyG alginate.

This result confirms what was suggested by the sequence and structural analysis, that the loops surrounding the active site and the residues in the binding pocket far away from the active site play a role in substrate recognition.

### Characterization of the alginate lyase activity of the candidate proteins on *P. aeruginosa* biofilms

**Figure 4:**
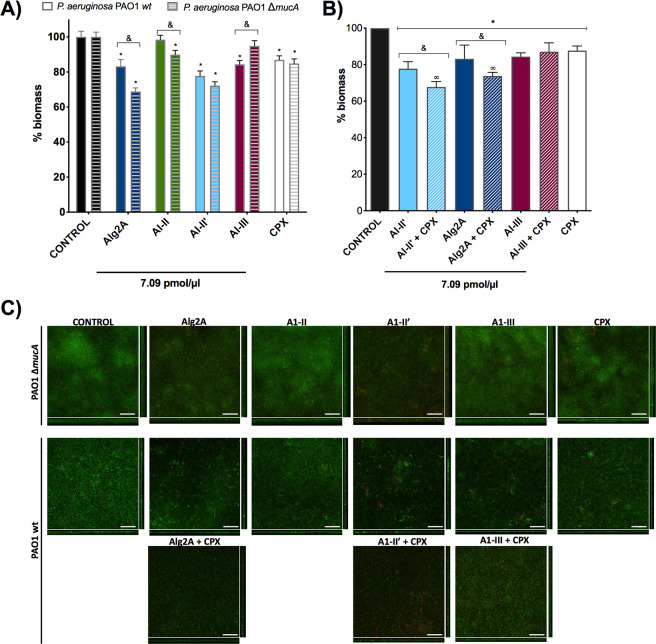
Biofilm degradation activity. (**A**) The histogram shows the ability of different enzymes to degrade biofilms formed by *P. aeruginosa* PAO1 *wt* (solid bars) and PAO1Δ*mucA* (striped bars). The concentration of enzyme was the same in each sample (7.09 pmol/µl), that corresponds to 0.32 mg/ml for Alg2A; 0.14 mg/ml for A1-II; 0.22 mg/ml for A1-II’; and 0.23 mg/ml for A1-III. The values are normalized against the control sample (non-treated biofilm) that has 100% biomass. The decrease in the percentage of biomass reflects the effect of the treatment. The asterisk (*) indicates a significant difference (p < 0.05) when the sample is compared with the control. The (&) indicates a significant difference (p < 0.05) when the PAO1 *wt* strain is compared with PAO1Δ*mucA*. (**B**) Synergism between antibiotics and the AlyA1, A1-III and Alg2A alginate lyase proteins. The asterisk (*) indicates a significant difference (p < 0.05) when the sample is compared with the control. The (&) indicates a significant difference (p < 0.05) when alginase is compared with alginase + ciprofloxacin. The (∞) indicates a significant difference (p < 0.05) when the sample is compared with ciprofloxacin. (**C**) Sum of stack images and the corresponding orthogonal views of the confocal microscopy images of *P. aeruginosa* PAO1 *wt* and PAO1 Δ*mucA* biofilms treated with 7.09 pmol/µl of the different alginate lyases and 1 µg/ml of ciprofloxacin (CPX). Red and green colors show the *P. aeruginosa* dead and alive cells detected by the LIVE/DEAD staining kit, respectively. Scale bar corresponds to 50 µm.

The enzymatic activity of alginate lyases was evaluated in *P. aeruginosa* biofilms grown for 96 hours before treatment. The experiment was performed in flow cell chambers where disaggregation was followed by imaging with a confocal microscope (Fig. 4). After treatment with different alginate lyases for 12 hours, we measured the biomass, thickness and roughness of the biofilms formed by wild type *P. aeruginosa* PAO1 (non-mucoid) and PAO1Δ*mucA* (mucoid strain) (Table S9). By measuring the difference in biofilm biomass between the untreated and treated samples, we were able to estimate the level of biofilm degradation by different alginate lyases (Fig. 4). After 12 hours of treatment, the Alg2A and A1-II’ proteins resulted in the highest reduction of biofilm biomass (around 30%) of both *P. aeruginosa* strains, confirming their degradation activity (Fig. 4A). Interestingly, Alg2A was more efficient at degrading the biofilm of PAO1Δ*mucA* than that of PAO1 *wt*. In contrast, A1-II’ degraded the biofilms formed by both strains to a similar extent. Although some biomass decrease was observed for A1-II in the PAO1 *∆mucA* strain biofilm, the same alginate lyase had no reduction activity in the PAO1 *wt* biofilm. In the case of the A1-III alginate lyase, the reduction of the biomass was only observed in the PAO1 *wt* strain. However, the activity of these two enzymes (A1-II and A1-III) was lower when compared to the activity of the rest of the studied proteins (Alg2A and A1-II’). Confocal microscopy images corroborated the abovementioned decreases in biomass (Fig. 4C, Supplementary Fig. S4). The ability of these enzymes to degrade biofilms was also compared with ciprofloxacin (CPX) antibiotic treatment, a benchmark treatment that results in approximately 15% biofilm reduction (Fig. 4A). After 12 hours of biofilm formation, A1-II’ is significantly more efficient at degrading the biofilm than pure CPX treatment.

In summary, we have shown that the broad spectrum polyM/G Alg2A and A1-II’ alginate lyases (PL7 family), but not the polyM or polyG alginate lyases like A1-II, (PL7) A1-III (PL5) and AlyA1 (PL7), can dissolve biofilms formed by *P. aeruginosa*.

### Synergistic effects between alginate lyases and antibiotic treatment in the degradation of biofilms

To gain insight into the role of alginate lyase activity in enhancing the effect of antibiotics, we treated *P. aeruginosa* PAO1 *wt* biofilms with Alg2A, A1-III or A1-II’ together with CPX and studied the impact on biofilm biomass disaggregation. As shown in Fig. 4B, the combination of A1-II’ or Alg2A with CPX produced a synergistic effect on biofilm disaggregation, with more than a 10% increase in the reduction of the biofilm biomass. Therefore, as previously shown, the combination of A1-III with CPX did not produced a synergistic effect (Fig. 4B). Thus, degradation of biofilm by enzymes with polyM/G activity is necessary to see an effect by CPX.

We discarded any antimicrobial activity by A1-II’ and Alg2A because no effect on the viability of PAO1 *wt* and PAO1Δ*mucA* strains was observed when grown in planktonic culture (Supplementary Fig. S3). Altogether, these results reveal that using Alg2A or A1-II’ alginate lyases to dissolve *P. aeruginosa* biofilms enhance antibiotic treatment.

## Discussion

Alginate, one of the main virulence factors associated with *P. aeruginosa* biofilms, has been the main target over the last decades when attempting to uncover alternative treatments for CF. The alginate heterogeneity generated by different combinations of β-D-mannuronate (M) and α-L-guluronate (G), as well as their different acetylation profiles, make this exopolysaccharide very difficult to target.

Many studies demonstrating the antibiofilm activity of alginases have used crude cell extracts from *P. aeruginosa*^20,23,45^. Other groups used purified enzymes such as A1-III and AlgL, and concluded that the synergistic effects with antibiotics were independent of the enzyme activity^47^.

Upon testing the ability to degrade the biofilms of two different *P. aeruginosa* strains (one of them mucoid), by different alginases, representing the two major families and with different substrate specificities, we consistently found that the broad spectra alginate lyases were the most active enzymes. In fact, only when polyM/G enzymes were combined with ciprofloxacin antibiotic, the synergistic effect was observed. In contrast, enzymes which only have polyG and polyM degradation activities respectively, showed less activity towards the biofilms and no synergistic effect with CPX. This agrees with previous results that showed polyG that A1-III did not amplifly the effect of antibiotics against *P. aeruginosa* biofilms.

We also found that among polyM/G enzymes, A1-II’ was able to degrade the brown seaweed substrate more efficiently than Alg2A. Altogether, these results suggest that A1-II’ and Alg2A have a broad activity spectrum against different biofilms because they degrade a greater variety of alginate matrixes. In fact, a different endolytic reaction mode was assigned to the Alg2A enzyme because it produces a larger amount of penta-, hexa-, and hepta-saccharides from the hydrolysis products when compared with other alginate lyases.

In conclusion, we have unraveled the effect that different alginate lyases have on *P. aeruginosa* biofilm degradation and we have identified Alg2A and A1-II’ as good disperal agents. We show an increase in the efficiency of antibiotic treatment when combined with Alg2A or A1-II’. Characterization of different alginate lyase proteins on biofilms reveled that Alg2A and A1-II’, despite having similar polyG/M and different activity against seaweed brown algynate, are the best candidates to use for degrading biofilms formed by different *P. aeruginosa* strains.

## Methods

### Ionic exchange and gel filtration chromatography

For anion exchange chromatography we used a HiTrap Q HP column (GE Healthcare product # 17-1153-01) in conjunction with an ÄKTA-FPLC system. The alginate lyase sample (Sigma #A1603) was dissolved in 20 mM Tris pH 8 as buffer A and applied to the pre-equilibrated column. As buffer B, we used the same buffer with 1 M NaCl. After 20 ml, we increased the salt concentration to 500 mM over 50 ml and collected fractions of 1 ml.

For gel filtration chromatography a Superdex-75 column was used (GE Healthcare product # 17-5174-01) with 20 mM Tris pH 8 and 150 mM NaCl as running buffer. The fraction size was 0.5 ml.

To achieve the presented resolution of proteins in the crude extract, we first performed an anion exchange chromatography, pooled fractions 37–42, 48–51 and 55–60, concentrated them and performed subsequent gel filtration. After the chromatographic runs, all fraction were supplemented with 10% glycerol to ensure protein stability.

### Mass spectrometry of fractions

All experimental proteomics procedures were performed by the UPF/CRG Proteomics Unit. We performed an initial tryptic digestion and peptide fingerprint MS analysis on the crude alginate lyase extract (Sigma A1603). Our aim was to screen for known toxins or other proteins with biofilm dissolving properties. We did not yield any reasonable lead from this. Therefore, we cut the protein bands identified as peaking using the halo activity test from an SDS gel. The bands were digested by trypsin or chymotrypsin and analyzed by nano-LC MS/MS. The resulting peaks were analyzed as DeNovo assembly using the software PEAKS (Bioinformatics solutions Inc.).

### Bacterial strains

Wild type *P. aeruginosa* PAO1 (ATCC 15692) and its isogenic *mucA* mutant PAO1Δ*mucA* strain (lab strain) were grown in LB medium at 37 °C. As standard conditions, *E. coli* BL21 and TOP10 strains were cultured at 37 °C in LB with the respective antibiotics added (50 µg/ml kanamycin).

### Proteins expression and purification

Synthetic genes encoding for Hypothetical protein (WP_012026046.1), Alg2A (AEB69783.1), AlyA1 (WP_013992548.1), A1-II (AGJ83952.1), A1-III (BAB03312.1) and A1-II´ (BAD16656.1) were ordered from GenScript. Then, they were cloned using Gibson assembly into pETM14, which results in a His-tag on the C-terminus of the protein. Expression was performed in *E. coli* BL21 and induction was performed at 16 °C with 1 mM of IPTG overnight. Two purification steps were performed: a nickel affinity chromatography followed by a desalting PD10 column, both in the 50 mM Tris pH 7.4, 300 mM NaCl, 10% glycerol, and 2 mM DTT. Proteins were analyzed by SDS gel electrophoresis.

### Halo test assay

For the halo test the *P. aeruginosa* PAO1 *wt* cells were grown on agar plates with *Pseudomonas* isolation medium (PI medium) (Bactopeptone 20 g/l, MgCl_2_ 1.4 g/l, K_2_SO4 10 g/l, glycerol 1%, pH 7.2). First the Petri dish was seeded with 1 ml of a bacterial culture (OD_600_=0.8) and paper disks (MDB-oxoid CT0998-B) were placed on the agar. Then, the disks were soaked with 10 µl of alginate lyase solution (17.5, 8.75 and 4.375 pmols) and the plates incubated for 16 hours at 37 °C. Formation of halos was then evaluated by visual inspection and direct measurement of halo diameter. Image J program was used to calculate the diameter of halos obtained from pictures and measured in pixels.

### Measurements of activity by UV assay

Activity of the alginate lyase was determined by the increase in absorbance at 235 nm due to the formation of a carbon-carbon double bond at the end of the product generated from lyase-mediated cleavage of alginate. Three different substrates were tested: brown seaweed alginate (Sigma# W201502 A straight-chain, hydrophilic, colloidal, polyuronic acid composed of glucuronic and mannuronic acid residues), ELICITYL # DP25-DP45 Guluronate oligosaccharides (polyG) and ELICITYL # DP20-DP35 Mannuronate oligosaccharides (polyM).

The substrates were dissolved in a solution of 20% glycerol and 20 mM Tris pH 7.4 so as to reach a final alginate concentration of 0.2%. Then, 50 µL of these substrates were added to the wells containing 7.1 pmol of each enzyme. As positive control we used 10 pmols of Sigma A1603 alginase. Absorbance was measured at 235 nm every 2 minutes for 78 minutes using UV-star microplates, 96wells, (Greiner #655801).

### Biofilm cultivation in flow cell chambers and microscopy

*P. aeruginosa* biofilms were grown in LB medium supplemented with 0.2% glucose at 20 °C in flow cell chambers as was previous described^56,57^. After 96 h the mature biofilm was treated with different alginate lyases for 12 h. For analysis, the biofilm was stained using the LIVE/DEAD BacLight Bacterial Viability kit (Thermo Scientific) and visualized with a Zeiss LSM 800 confocal laser scanning microscope (CSLM) using the 20×/0.8 objective with excitation wavelengths of 488 and 560 nm. Microscope images were processed with the ImageJ analysis software and COMSTAT 2, a specific biofilm analysis software was used to quantify the biofilm biomass, thickness and roughness^58^.

### Antimicrobial activity of alginate lyases

*P. aeruginosa* PAO1 *wt* and PAO1 *∆mucA* were grown until an optical density (OD_550_ nm) of 0.1 was reached and then plated in a microtiter plate (Corning 3596 Polystyrene Flat Bottom 96 Well Corning, NY, USA). The different alginases were added at a similar concentration to the one used in the biofilm analysis (7.09 pmol/µl). Bacterial growth at 37 °C and 150 rpm was monitored for 8 hours, and the absorbance measured at 550 nm every 15 minutes in an SPARK Multimode microplate reader (Tecan, Männedorf, Switzerland).

### Statistical analysis

Values are expressed as mean ± standard deviation (SD). Statistical analyses were performed using GraphPad Prism 6.00 (GraphPad Software, San Diego, CA, USA) software package. Single comparisons were performed with Welch’s correction. A value of *p* < 0.05 was considered as statistically significant.

## Supplementary information

Supplementary information.

Supplementary tables
