# Supplementary material for: Characterization of different alginate lyases for dissolving Pseudomonas aeruginosa biofilms
Source: Sci Rep. 2020 Jun 10;10:9390. doi: 10.1038/s41598-020-66293-2 (PMC7287115; doi:10.1038/s41598-020-66293-2)
Supplement: Supplementary file 1 — Supplementary information. [file 41598_2020_66293_MOESM1_ESM.docx]

**Characterization of different alginate lyases for dissolving *Pseudomonas aeruginosa* biofilms**

Núria Blanco-Cabra^1*^, Bernhard Paetzold^2*^, Tony Ferrar^3^, Rocco Mazzolini^3^, Eduard Torrents^1^, Luis Serrano^3,4,5,&^, Maria LLuch-Senar^3,&^

**SUPPLEMENTARY FIGURES AND TABLES**

**Figure S1. Identification of alginate lyases by MS.** Peptides associated with AlyA1 and Alg2A proteins, identified by MS are labeled in blue in the amino acid sequences of the proteins.

**
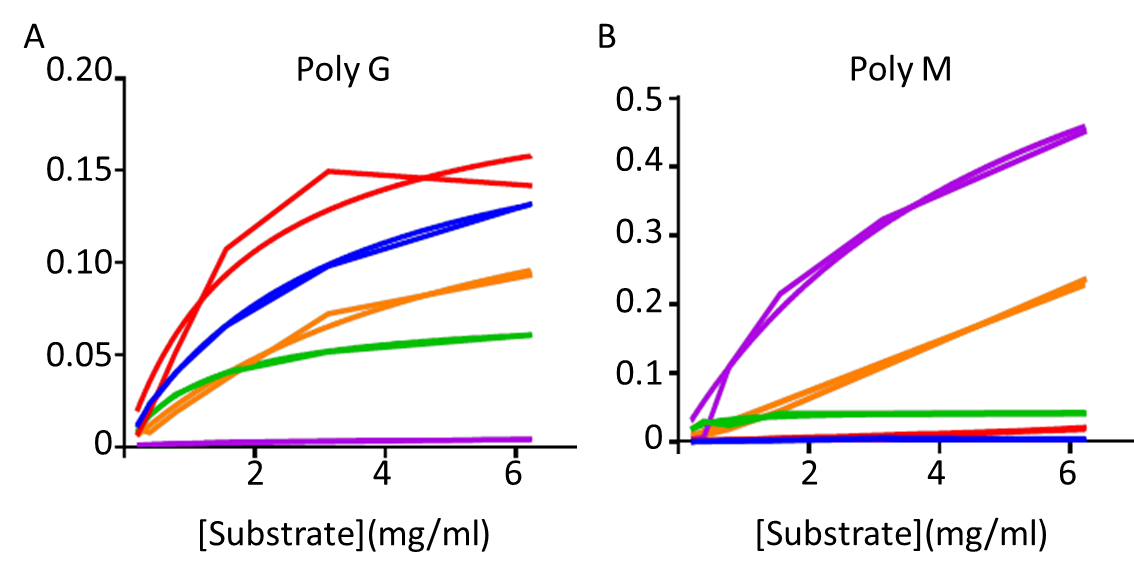
Figure S2. Michaelis–Menten saturation curves for the different alginate lyases reactions.** The A and B graphs show the relation between the substrate concentration and reaction rate for Poly G and Poly M substrates, respectively. Different colors represent the curves adjusted for different enzymes: AlyA1 (blue), A1-II (red), A1-II’ (green), A1-III (violet) and Alg2A (orange).


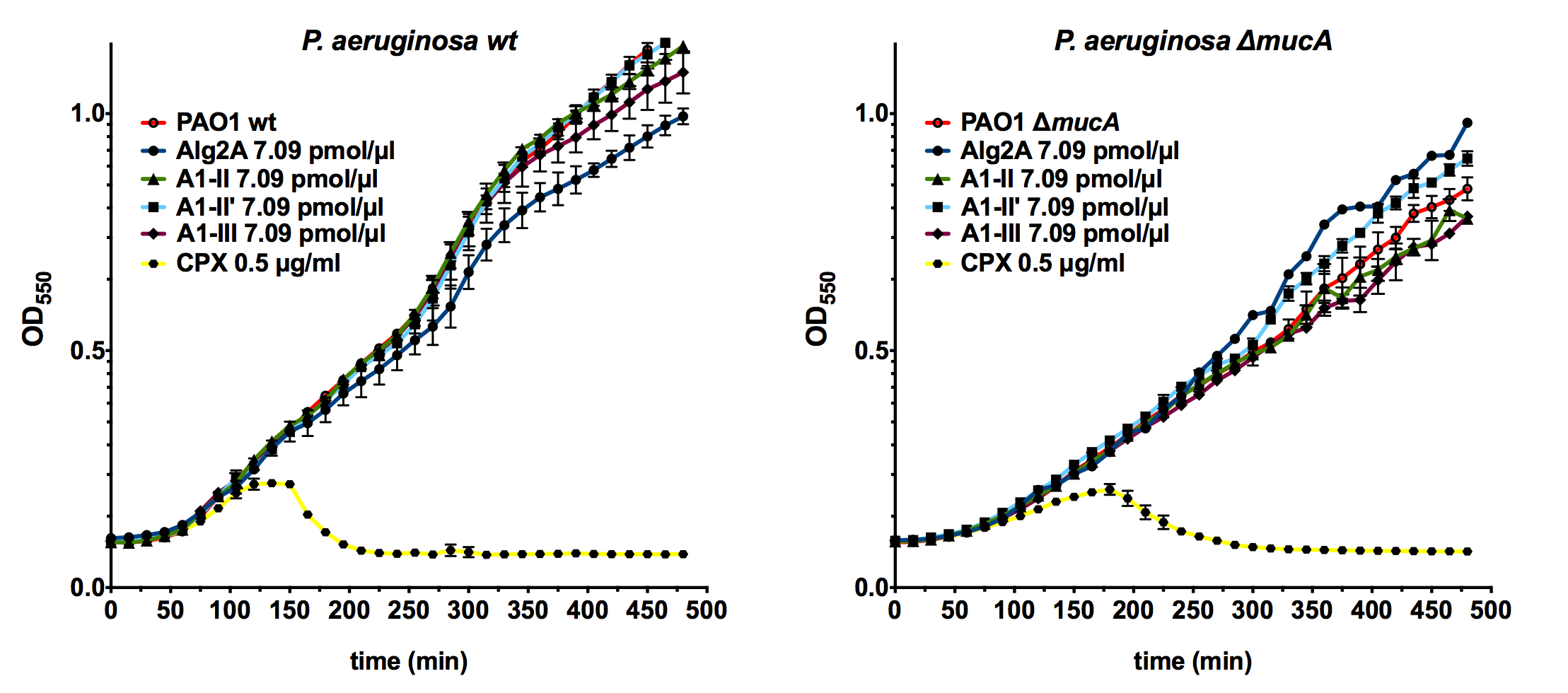
**Figure S3. Growth curves of *P. aeruginosa* PAO1 wt and PAO1Δ*mucA* strains treated with different alginate lyases.** Antimicrobial activity of different alginate lyases was tested by studying the growth effect of adding the different proteins at 7.09 pmol/µl after 8 hours of growth. No effect in the exponential growth of different strains was observed. As a control, ciprofloxacin was added at 0.5 µg/ml.

**Figure S4.** Sum of stack images and the corresponding orthogonal views of the confocal microscopy images of *P. aeruginosa* PAO1 *wt* and PAO1 Δ*mucA* 92-hour biofilms treated for 12 hours with 7.09 pmol/µl of the different alginate lyases and 1 µg/ml of ciprofloxacin (CPX). Red and green colors show the *P. aeruginosa* dead and alive cells detected by the LIVE/DEAD staining kit, respectively. Scale bar corresponds to 50 µm.

**Table S9. Values of confocal microscopy assays:** Values of biomass, thickness and roughness ± SD
